# Genetic and Clinical Profiles of Disseminated Bacillus Calmette-Guérin Disease and Chronic Granulomatous Disease in China

**DOI:** 10.3389/fimmu.2019.00073

**Published:** 2019-01-29

**Authors:** Tao Li, Xian Zhou, Yun Ling, Ning Jiang, Jingwen Ai, Jing Wu, Jiazhen Chen, Li Chen, Xiaowen Qian, Xuhui Liu, Xiuhong Xi, Lu Xia, Xiaoyong Fan, Shuihua Lu, Wen-Hong Zhang

**Affiliations:** ^1^Department of Infectious Diseases, Huashan Hospital, Fudan University, Shanghai, China; ^2^Shanghai Public Health Clinical Center, Fudan University, Shanghai, China; ^3^School of Life Sciences, Fudan University, Shanghai, China; ^4^Department of Medical Microbiology and Parasitology, Fudan University, Shanghai, China; ^5^Children's Hospital of Fudan University, Shanghai, China

**Keywords:** Bacillus Calmette-Guérin, disseminated BCG disease, BCGosis, vaccination, chronic granulomatous disease, complications

## Abstract

**Background:** Disseminated Bacillus Calmette-Guérin disease (D-BCG) in children with chronic granulomatous disease (CGD) can be fatal, while its clinical characteristics remain unclear because both diseases are extremely rare. The patients with CGD receive BCG vaccination, because BCG vaccination is usually performed within 24 h after delivery in China.

**Methods:** We prospectively followed-up Chinese patients with CGD who developed D-BCG to characterize their clinical and genetic characteristics. The diagnoses were based on the patients' clinical, genetic, and microbiological characteristics.

**Results:** Between September 2009 and September 2016, we identified 23 patients with CGD who developed D-BCG. Their overall 10-year survival rate was 34%. We created a simple dissemination score to evaluate the number of infected organ systems and the survival probabilities after 8 years were 62 and 17% among patients with simple dissemination scores of ≤3 and >3, respectively (*p* = 0.0424). Survival was not significantly associated with the CGD stimulation index or interferon-γ treatment. Eight patients underwent umbilical cord blood transplantation and 5 of them were successfully treated. The genetic analyses found mutations in *CYBB* (19 patients), *CYBA* (1 patient), *NCF1* (1 patient), and *NCF2* (1 patient). We identified 6 novel highly likely pathogenic mutations, including 4 mutations in *CYBB* and 2 mutations in *NCF1*.

**Conclusions:** D-BCG is a deadly complication of CGD. The extent of BCG spreading is strongly associated with clinical outcomes, and hematopoietic stem cell transplantation may be a therapeutic option for this condition.

## Introduction

Bacille Calmette-Guerin (BCG), an attenuated strain of *Mycobacterium bovis*, was originally isolated from a cow with tuberculosis. The BCG vaccine was first used to immunize humans in 1921 (1). With protective effect against life-threatening tuberculosis(TB) such as tuberculosis meningitis and disseminated TB in children, BCG vaccination is recommended in countries with a high TB burden (2). It is one of the most widely used of all current vaccines and over 150 countries have universal BCG vaccination program (3). Although more than 14 substrains of BCG have been used as BCG vaccine strains in different parts of the world differ in terms of their genetic and phenotypic properties, commonly administered BCG vaccine strains induce comparable protective immunity against TB (1).

Disseminated Bacillus Calmette-Guérin (D-BCG) disease is the most fatal adverse event after BCG vaccination, and is associated with a mortality rate of 60–80% (4–6). Early studies have indicated that acquired or inherited immunodeficiency can predispose the patient to infection with an attenuated strain of *M. bovis* BCG (7). Among them, chronic granulomatous disease (CGD) is one of the representative (8). Patients with CGD suffer from a variety of recurrent bacterial and fungal infections (9). It is caused by an inherited defect in the nicotinamide adenine dinucleotide phosphate (NADPH) oxidase enzyme complex that consists of two membrane-spanning subunits, gp91^*phox*^ and p22^*phox*^, as well as three cytosolic components p47^*phox*^, p67^*phox*^, and p40^*phox*^ (10). Mutations within the X-linked gp91 ^*phox*^ gene (*CYBB*) cause the X-linked form of CGD, while the autosomal recessive forms of CGD are due to mutations in in *CYBA, NCF1, NCF2*, or *NCF4* which encode for p22 ^*phox*^, p47 ^*phox*^, p67 ^*phox*^, and p40 ^*phox*^, respectively (10).

BCG vaccination is contraindicated for persons with congenital cell-mediated or severe combined immunodeficiency, immunodeficiency syndromes (e.g., HIV/AIDS, known or suspected congenital immunodeficiency, leukemia, lymphoma, or other malignant disease) (2). Because patients with CGD have impaired reactive oxygen species [ROS]-producing phagocyte NADPH oxidase, which may weaken their anti-mycobacterial defense (8), CGD is one of the contraindications of BCG vaccination. However, as BCG is usually vaccinated very early in infancy, there is not enough time to screen for CGD and other vaccination contraindications. Many reports have described minor BCG complications, such as regional lymphadenitis, in CGD cases (11–16), D-BCG associated with CGD is extremely rare (14, 17), as the incidences of D-BCG and CGD are ~2 cases per 1 million vaccinations (18) and 5 cases per 1 million live births (19), respectively. This manuscript describes the genetic and clinical characteristics of 23 Chinese patients with D-BCG associated with CGD.

## Materials and Methods

### Case Definition

CGD was diagnosed based on the patient's clinical presentation and confirmed using either the dihydrorhodamine 123 (DHR) oxidation assay or genetic analysis. DHR assay is tested using FACScam(Becton Dickinson, CA, USA) with methods established by Yu et al. (20). We detected *CYBB* mutation by PCR using synthetic oligonucleotide primers designed to amplify the *CYBB* gene. For patients with *CYBB* mutation negative, we performed whole exon sequencing. D-BCG was classified according to the criteria in Table 1. A simple dissemination site-based score (1 point per involved organ system) was used to evaluate the degree of dissemination to the lungs, bones, liver, spleen, kidney, heart or pericardium, gastrointestinal tract, peritoneum, nose, ears, skin, and soft tissues. The scoring was performed by attending physicians based on the results of the clinical examination, imaging, and laboratory testing.

**Table 1:** The diagnostic criteria and classifications for Bacillus Calmette-Guérin (BCG) disease.

**DIAGNOSTIC CRITERIA**
1. A history of BCG vaccination: vaccination record showing BCG vaccination. 2. Evidence of dissemination: evidence of BCG infection at the presentation that involved ≥1 anatomical site in addition to that of the vaccination site and its regional lymph nodes. 3. Pathology/etiology: clinical specimen that was positive for *Mycobacterium tuberculosis* complex based on culturing, acid-fast staining, and histological detection of caseating granuloma. 4. BCG identification: real-time PCR (careTB PCR ASSAY kit, Qiagen) was performed to identify the *M. tuberculosis* complex according to the manufacturer's instructions. Positive specimens were subsequently testing using a BCG assay based upon genomic deletions at RD1, 9, and 10, which was established by Parsons et al. (21).
**DIAGNOSTIC CLASSIFICATIONS**
***Definite (all of the following criteria)***
History of BCG vaccination
Evidence of dissemination
Evidence regarding the pathology/etiology
BCG identification
***Highly probable (all of the following criteria)***
History of BCG vaccination
Evidence of dissemination
Evidence regarding the pathology/etiology
***Probable (all of the following criteria)***
History of BCG vaccination
Evidence of dissemination

### Data Collection

We conducted a prospective observational study to follow up patients who had clinical symptoms of D-BCG and were hospitalized at the Shanghai Public Health Clinical Center of Fudan University from September 1, 2009, to September 1, 2016. All patients and/or their guardians provided written informed consent to be followed-up. This study's observational protocol was approved by the ethics committee of the hospital. The patients were diagnosed and categorized based on the criteria that are listed in Table 1, and their clinical, radiological, pathological, and microbiological characteristics.

### Statistical Analysis

All statistical analyses were performed using GraphPad Prism (version 6.0) and IBM SPSS software (version 22.0). Categorical and continuous variables were compared using Fisher's exact test and the Mann–Whitney *U*-test, respectively. Kaplan–Meier survival curves were compared between the groups using the log-rank test. All tests were two-sided, and differences were considered statistically significant at *p* < 0.05.

## Results

Between September 2009 and September 2016, 78 patients were diagnosed with D-BCG at our center. Among them, 23 patients were diagnosed with CGD (Table 2). The diagnosis of CGD was based on DHR testing in 1 patient, genetic analysis in 6 patients, and a combination of DHR testing and genetic analysis in 16 patients. The diagnoses of D-BCG in CGD patients were definite in 6 patients, highly probable in 9 patients, and probable in 8 patients. All 23 patients were male, and their median age at referral to our hospital was 12.2 months (mean: 25.7 months, range: 2.9–123.7 months). The children were from 23 different families that resided in 10 Chinese provinces (mostly in the southeast region). All patients had undergone BCG vaccination with strain D_2_PB302 at the left deltoid region within 24 h after birth, based on the national vaccination policy and protocol.

**Table 2:** Patient characteristics.

	***N***
**DISTRIBUTION OF GENETIC MUTATIONS**	
*CYBB*	19
*CYBA*	1
*NCF1*	1
*NCF2*	1
**DISSEMINATED BCG DISEASE DIAGNOSIS**	
Definite	6
Highly probable	9
Probable	8
**FAMILY HISTORY**	
Early death of male sibling	4
Consanguine marriage	1
Recurrent spontaneous abortion	1
**FOLLOW-UP**	
Alive	11
Deceased	10
Lost to follow-up	2

*BCG, Bacillus Calmette-Guérin; CYBB, the beta chain of cytochrome b; CYBA, the alpha chain of cytochrome b; NCF1, neutrophil cytosolic factor 1; NCF2, neutrophil cytosolic factor 2*.

**Figure 1:**
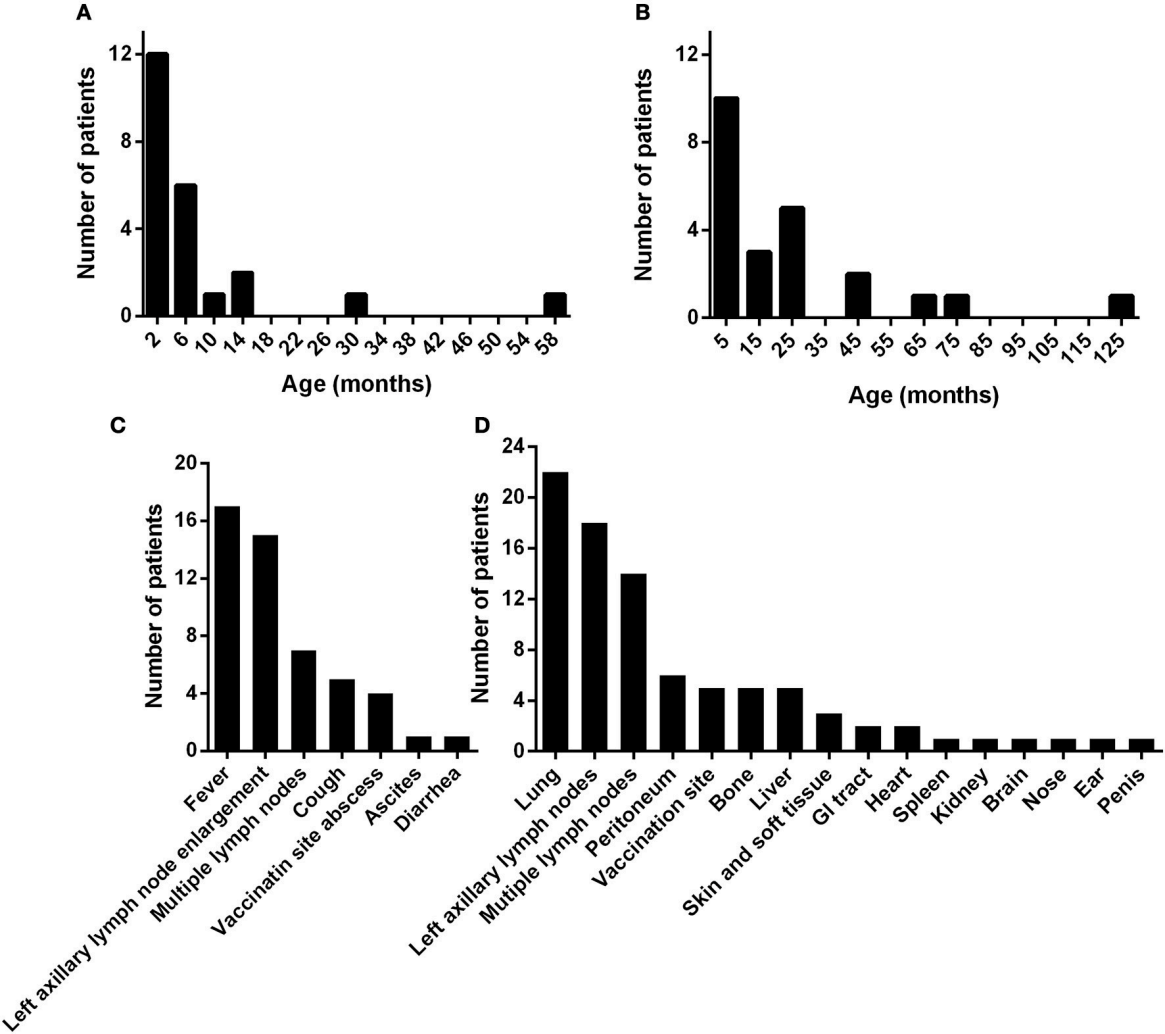
The ages at symptom onset **(A)** and at the diagnosis of disseminated Bacillus Calmette-Guérin disease **(B)** in 23 patients with CGD. The **(C)** initial symptoms, and **(D)** dissemination sites of Bacillus Calmette-Guérin disease. GI, gastrointestinal.

The median age at the onset of BCG disease was 3.0 months (mean: 7.6 months, range: 0.3–58.5 months) (Figure 1A). Nineteen of the 23 children (82.6%) had symptoms of BCG disease before the age of 1 year. The median age at the diagnosis of D-BCG was 12.4 months (mean: 24.7 months, range: 2.9–123.1 months) (Figure 1B). The median lag between the onset and diagnosis of BCG disease was 5.4 months (mean: 17.2 months, range: 0.6–121.1 months). Seventeen of the 23 children (73.9%) developed symptoms of BCG disease as the initial symptom of CGD. The median age at the diagnosis of CGD was 12.6 months (mean: 23.9 months, range: 1.3–121.1 months), and 13 of the 23 patients had been diagnosed with D-BCG before developing CGD.

The local adverse reactions to the BCG vaccine included left axillary lymph node enlargement (*n* = 15, 65%) and an abscess at the vaccination site (*n* = 4, 17%). The initial symptoms of D-BCG were fever (*n* = 17, 74%), multiple lymph node enlargement (*n* = 7, 30%), cough (*n* = 5, 22%), ascites (*n* = 1, 4%), and diarrhea (*n* = 1, 4%) (Figure 1C). As the infection deteriorated, various organs developed signs of BCG dissemination (Figure 1D). Most patients (*n* = 22, 96%) had pulmonary involvement, 14 patients (61%) had multiple systematic lymph node enlargement, and abdominal involvement was common (ascites: 6 patients, liver involvement: 5 patients, gastrointestinal tract involvement: 2 patients, splenomegaly: 1 patient, and hydronephrosis: 1 patient). Five patients (22%) had bone lesions, 3 patients had BCG dissemination to the skin or soft tissues beyond the vaccination site, and 2 patients had BCG dissemination to the pericardium. We also observed dissemination to the central nervous system, nose, ears, and penis (1 patient each). The average simple dissemination score was 2.8 affected organ systems (range: 1–6 organ systems).

**Figure 2:**
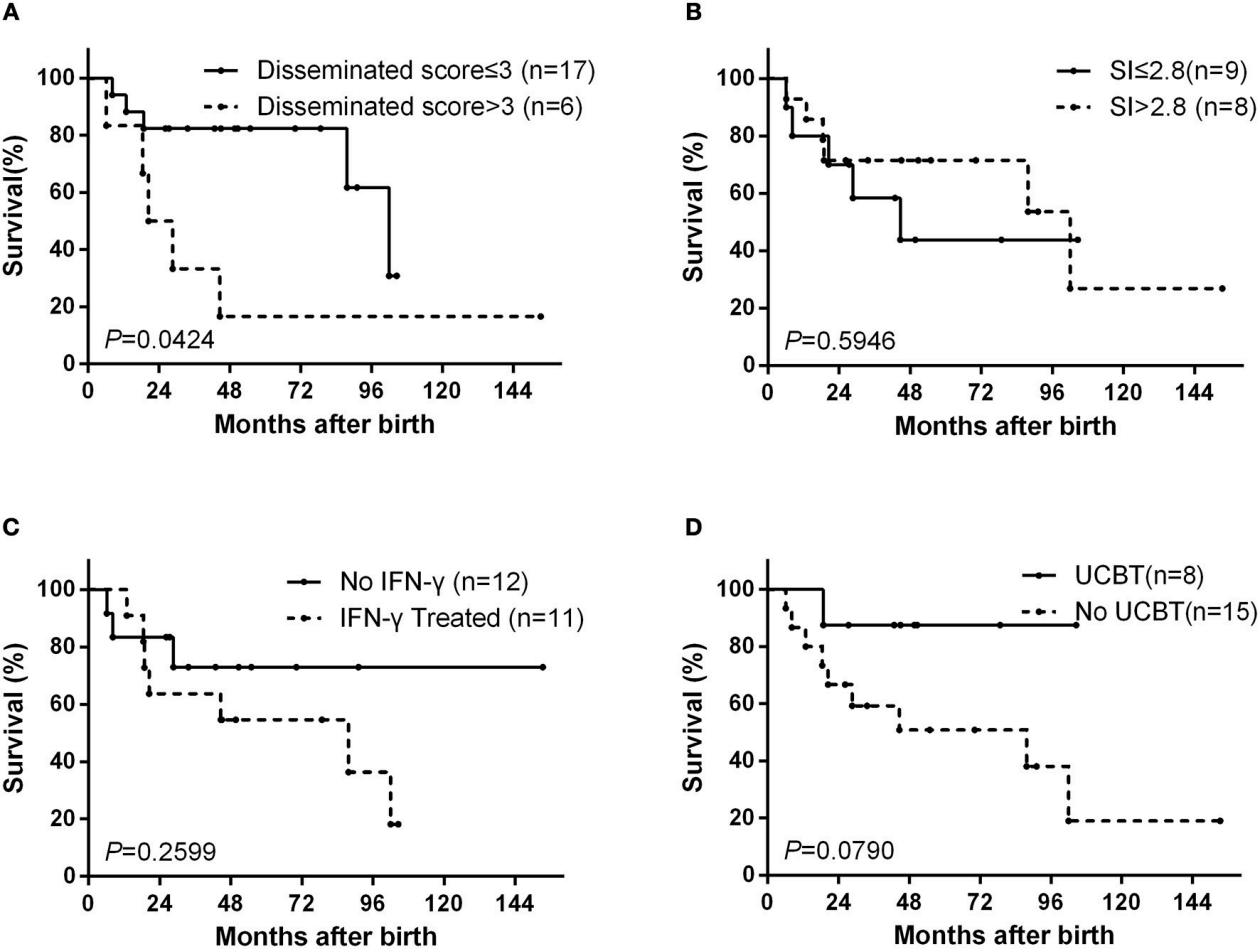
Kaplan–Meier survival curves from the date ot birth. The curves are shown for **(A)** according to dissemination score, **(B)** according to the dihydrorhodamine assay stimulation index (SI), **(C)** according to interferon (IFN)-γ treatment status, and **(D)** according to umbilical cord blood transplantation (UCBT) status.

At the last follow-up (April 21, 2018), 10 patients (44%) had died from complications of D-BCG at a median age of 21.3 months (mean: 35.2 months, range: 6.2–102.0 months), 11 patients (48%) were alive, and 2 patients (9%) had been lost to follow-up. The overall 10-year survival rate was 34% (Figure S1A). The survival probabilities after 8 years were 62% among patients with a dissemination score of ≤3 and 17% among patients with a dissemination score of >3 (*p* = 0.0424) (Figure 2A).

Among the 17 patients that underwent DHR testing, the median stimulation index (SI) was 2.79 (mean: 5.31, range: 1.5–18.4). There was no significant difference in the SI values when we compared deceased and surviving patients (Mann–Whitney test, *p* = 0.9336). The survival probabilities after 8 years were 44% for patients with an SI of ≤2.8 and 54% for patients with an SI of >2.8 (*p* = 0.5946) (Figure 2B).

In addition to the BCG infection, 14 patients (61%) had other infections that were caused by a virus, fungus, or bacterium. Six patients had ≥2 infections, and 7 patients had disseminated infection caused by chronic active Epstein-Barr virus infection (2 patients), blood culture-confirmed bacterial infections (4 patients), and autopsy-confirmed tuberculosis infection (1 patient). The most frequently isolated organisms were *Klebsiella pneumonia* and *Aspergillus* spp., and the lungs were the most frequently involved organs.

After the diagnosis, all patients received anti-BCG treatment, which was generally based on a regimen of isoniazid, rifampin, and ethambutol. Some patients received alternative treatment because of isoniazid resistance (1 patient), adverse reactions (5 patients), or clinical experience. As the subtle changes in the anti-BCG regimen were too complicated to analyze throughout the long-term treatment, we have listed the regimens in Table 3.

**Table 3:** The cohort's clinical and genetic characteristics.

**Patient no**.	**Province**	**CGD diagnosis**	**Mutation**	**Amino acid change**	**SI**	**BCG diagnosis**	**Other infections**	**Anti-BCG treatment**	**Follow-up**
1	Zhejiang	Genetic	*CYBA*^∧^			Probable	Epstein-Barr virus	HELfx	Alive (stopped anti-BCG treatment)
2	Hubei	DHR/genetic	*NCF2* c.304C>T (22)	p.(Arg102^*^)	2.13	Definite	–	HRZE	Deceased
3	Henan	DHR/genetic	*CYBB* c.466G>A (22)	p.(Ala156Thr)	18	Definite	*Pseudomonas putida, Klebsiella pneumoniae*	EAmkLzd	Deceased
4	Henan	Genetic	*CYBB* c.1514T>C (23)	p.(Leu505Pro)		Definite	*Aspergillus* spp., Epstein-Barr virus	HRZE	Deceased
5	Zhejiang	DHR/genetic	*CYBB* ^#^c.277C>T	p.(Gln93^*^)	2.2	Definite	Mumps virus	HRE	Deceased
6	Jiangsu	DHR/genetic	*CYBB* ^#^c.337_338del	del exon 5? p.(Ala113fs)?	2.43	Definite	Fungal	HRE	Alive (stopped anti-BCG treatment)
7	Fujian	Genetic	*CYBB* c.935T>A (13)	p.(Met312Lys)		Definite	–	HR	Alive (continuing anti-BCG treatment)
8	Henan	DHR/genetic	*CYBB* ^#^c.1150_1151del	del exon 9? p.(Lys384fs)?	1.56	Probable	*Klebsiella pneumoniae*	HERLzd	Deceased
9	Zhejiang	DHR/genetic	*CYBB* ^#^c.1151+1_1151+2del	del exon 9?	4.54	Highly probable	–	HRE	Deceased
10	Fujian	DHR/genetic	*CYBB* c.1082G>T (24)	p.(Trp361Leu)	3.38	Highly probable	*Aspergillus terreus, Cellulomonas* spp.	ClrEMfx	Deceased
11	Shanghai	DHR/genetic	*CYBB*^∧^		2.79	Highly probable		HRE	Alive (stopped anti-BCG treatment)
12	Anhui	DHR/genetic	*CYBB*^∧^		18.4	Highly probable	*Staphylococcus aureus*, G+ cocci, G– cocci	HRE	Unkown
13	Anhui	DHR/genetic	*CYBB* c.1499A>G (25)	p.(Asp500Gly)	8.84	Highly probable	*Enterococcus faecium, Klebsiella pneumoniae, Escherichia coli*	HELfx	Deceased
14	Fujian	DHR/genetic	*CYBB*^∧^		11	Probable	*Salmonella typhimurium*	HRZ	Alive (stopped anti-BCG treatment)
15	Henan	Genetic	*NCF1* ^#^c.269G>A; ^#^c.761_798del	p.(Arg90His) p.(Val255fs)		Highly probable	–	HREPto	Alive (stopped anti-BCG treatment)
16	Jilin	DHR/genetic	*CYBB* c.1095del (26)	p.(Phe366fs)	2.3	Probable	–	HR	Alive (continuing anti-BCG treatment)
17	Jiangsu	DHR/genetic	*CYBB*^∧^		1.9	Highly probable	–	HR	Alive (continuing anti-BCG treatment)
18	Jiangxi	DHR/genetic	*CYBB*^∧^		1.5	Probable	G+ cocci	HERft	Alive (continuing anti-BCG treatment)
19	Hunan	DHR	N/A		2.31	Highly probable	*Candida* spp.	ELzdAmkPas	Deceased
20	Hunan	DHR/genetic	*CYBB* c.1082G>T (24)	p.(Trp361Leu)	2.85	Definite	*Mycobacterium chelonae* subsp. *M. abscessus; M. intracellulare; M. tuberculosis*	HR	Deceased
21	Zhejiang	Genetic	*CYBB*^∧^			Probable	–	HR	Unknown
22	Jiangsu	Genetic	*CYBB* c.1085C>T (26)	p.(Thr362Ile)		Probable	*Staphylococcus epidermidis*	HRE	Alive (continuing anti-BCG treatment)
23	Jiangsu	DHR/genetic	*CYBB* c.1165G>A (26)	p.(Gly389Arg)	4.15	Probable	–	HRE	Alive (stopped anti-BCG treatment)

∧ *The mutation was noted on the clinical report without site specifications*.

# *Novel mutation site*.

? *Amino acid change predicted at splice sites*.

* *Designates a translation termination codon. DHR, dihydrorhodamine; BCG, Bacillus Calmette-Guérin; CYBB, the beta chain of cytochrome b; CYBA, the alpha chain of cytochrome b; NCF1, neutrophil cytosolic factor 1; NCF2, neutrophil cytosolic factor 2; H, isoniazid; R, rifampicin; Z, pyrazinamide; E, ethambutol; Lfx, levofloxacin; Amk, amikacin; Lzd, linezolid; Pas, para-aminosalicylic acid; Mfx, moxifloxacin; Pto, protionamide; Rft, rifapentine*.

Interferon (IFN)-γ was added to the treatment protocol for 11 patients. The use of IFN-γ was not associated with the dissemination score (Fisher's exact test, *p* = 1.0000), and the survival probabilities after 8 years were not significantly different between patients who did and did not receive IFN-γ treatment (36 vs. 73%, *p* = 0.2599) (Figure 2C). Eight patients underwent umbilical cord blood transplantation (UCBT), with a success rate of 63% (5 cases). 3 of 5 children who underwent successful UCBT have stopped the anti-BCG treatment. One child died at 4 months after failed UCBT. The other 2 child failed UCBT is continuing the anti-BCG treatment and waiting for the second one. The survival probabilities after 8 years were 88% among patients with UCBT and 38% among patients without UCBT (*p* = 0.0790) (Figure 2D).

**Figure 3:**
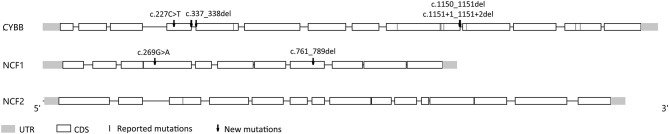
A schematic diagram of the mutations in our cohort.

Nineteen patients (82.6%) had X–linked recessive mutations in *CYBB gene*. One child's mother had a history of recurrent spontaneous abortion and 4 children had deceased brothers who died before the age of 1 year. Additionally, three children were identified with compound heterozygous or hemizygous mutations in other autosomal genes, including *CYBA* (1 patient), *NCF1* (1 patient), and *NCF2* (1 patient). A homozygous non-sense mutation (NM_000433.3: c.304C>T) was found in *NCF2* of one male, whose parents were consanguineous and carried the heterozygous mutation in this locus. Furthermore, we identified 6 novel highly likely pathogenic mutations, including 4 mutations in *CYBB* and 2 mutations in *NCF1* (Figure 3 and Table 3).

## Discussion

As both diseases have very low incidence rates, the clinical characteristics of D-BCG with CGD remains unclear. The present study evaluated 23 Chinese cases of D-BCG with CGD, which is, to the best of our knowledge, the largest reported cohort of these patients. The development of antifungal and antibiotic treatments has greatly decreased the mortality rate in conventionally treated CGD cases, and most patients can survive until their thirties (19, 27). However, the prognosis of patients with D-BCG and CGD remains poor. The 10-year survival rate in our cohort was 34%, which is consistent with the findings of previous studies (4, 18). Compared to D-BCG patients with unknown genetic defects, D-BCG patients with CGD required longer anti-BCG treatment (Figure S1B).

In the present study, 5 of 8 patients were successfully treated using UCBT, and 3 of 5 successful UCBT patients have stopped anti-BCG treatment, which indicates that UCBT may be a curative treatment for patients with CGD. Furthermore, compared to conventionally treated CGD, a previous study revealed that hematopoietic stem cell transplantation (HSCT) was associated with lower rates of infection and hospitalization (28). Although our small sample size limited the power of this analysis, we noticed an improved survival rate in this subgroup, and suggest considering early HSCT for patients with D-BCG and CGD, given the deadly effects of BCG dissemination. Unfortunately, the precise timing and conditioning intensity remain unclear for using HSCT to treat primary immunodeficiency (29), and this problem would be further aggravated among patients with BCG infection, given the lack of useful data.

Unlike relatively mild BCG complications that are usually limited to the regional lymph nodes (14, 30), our patients with D-BCG exhibited characteristic BCG dissemination to distant organs. Thus, we created a simple dissemination score to evaluate the number of infected organ systems, and found that it was both easy to measure and may help predict the prognosis in cases of D-BCG with CGD, which is quite bad for patients with score more than 3. In the present study, the BCG dissemination most commonly involved the lungs and systemic lymph nodes, with 22 of 23 cases exhibiting lung involvement and 13 of 23 cases exhibiting concurrent lung and systemic lymph node involvement.

More than 700 different mutations responsible for CGD have been identified (31), and we found 6 new mutations, 4 in *CYBB* and 2 in *NCF1*. Although we did not perform functional experiment, the clinical presentation, compromised granulocyte function with evidence of DHR assay and non-synonymous mutations in CGD-related genes were convicing evidence for causative mutations. However, the correlation between the genotype and the clinical presention, especially the occurrence of D-BCG in CGD patients is not clear. For BCG-vaccinated CGD patients, 8–56% of them had BCG lymphadenitis (11, 13, 15, 16, 27), 0–10% had D-BCG (11–13). The present study did not reveal a significant association between D-BCG and the DHR assay's SI value (residual NADPH oxidase function). This result is different from the findings of two previous studies (15, 32), but is consistent with the findings of Köker et al. in Turkey (15). Similar to China, Turkey has a national policy of BCG vaccination, and Köker et al. concluded that the residual NADPH oxidase activity of neutrophils was associated with later and less severe clinical presentation, although it did not affect the occurrence of BCG. Early studies have indicated that *CYBB* mutations (33) can cause a selectively impaired respiratory burst in macrophages and are associated with mendelian susceptibility to mycobacterial disease. Thus, the residual NADPH oxidase function of macrophages could be a useful predictor of prognosis among patients with both BCG and CGD.

The efficacy of IFN-γ for treating CGD remains controversial (34–37). The most cited study supporting the use of IFN-γ therapy was a randomized double-blind placebo-controlled trial of 128 patients with CGD, and revealed that IFN-γ substantially reduced the frequency of serious infections (34). However, a prospective multicenter study in Italy (37) found that long-term use of IFN-γ did not change the rate of infections per patient-year. In those studies, the IFN-γ was administered prophylactically and the primary endpoints were serious infections, while we evaluated treatment during the active infection period and survival outcomes. Therefore, additional studies are needed to provide better evidence regarding the efficacy of IFN-γ treatment for CGD.

Our study is limited by its observational nature and the small sample size, which limited the power of our statistics analyses. Furthermore, the sample size is too small to perform multivariate survival analysis. However, both D-BCG and CGD are extremely rare diseases, and the 23 cases in our cohort provide valuable information regarding the genetic and clinical characteristics of patients with both D-BCG and CGD.

## Conclusion

This study of 23 Chinese patients with D-BCG and CGD confirmed that D-BCG is a severe complication of CGD, and CGD patients is a subgroup of D-BCG patients with relatively poor prognosis. The extent of BCG spreading was strongly associated with clinical outcomes, although these outcomes were not associated with the residual NADPH oxidase function of neutrophils. Furthermore, IFN-γ therapy during active BCG infection was not associated with an improved survival rate. Based on our findings, we suggest that all patients with this fatal disease should be considered for early HSCT.

## Author Contributions

TL, XZ, YL, SL, and W-HZ: conception or design of the work; TL, XZ, XQ, LX, XL, and XX: data collection and patient care; TL, XZ, NJ, JA, JW, JC, and XF: data analysis and interpretation; TL and XZ: drafting the article. YL, JW, LC, and W-HZ: critical revision of the article; TL, XZ, YL, NJ, JA, JW, JC, LC, XQ, XL, XX, LX, XF, SL, and W-HZ: Final approval of the version to be published.

### Conflict of Interest Statement

The authors declare that the research was conducted in the absence of any commercial or financial relationships that could be construed as a potential conflict of interest.

## Abbreviations

BCG: Bacille Calmette-Guerin
CGD: chronic granulomatous disease
D-BCG: Disseminated Bacillus Calmette-Guérin disease
DHR: dihydrorhodamine 123
HSCT: hematopoietic stem cell transplantation
IFN: interferon
NADPH: nicotinamide adenine dinucleotide phosphate
SI: stimulation index
TB: tuberculosis
UCBT: umbilical cord blood transplantation
